# X-ray beam monitoring and wavelength calibration using four-beam diffraction

**DOI:** 10.1107/S1600577521012352

**Published:** 2022-01-01

**Authors:** XianRong Huang, Xianbo Shi, Lahsen Assoufid

**Affiliations:** a Advanced Photon Source, Argonne National Laboratory, 9700 South Cass Avenue, Lemont, IL 60439, USA

**Keywords:** X-ray energy calibration, beam position monitoring, multiple-beam diffraction, dynamical theory, synchrotron light source

## Abstract

Rigorous dynamical theory calculations show that four-beam diffraction (4BD) can be activated only by a unique photon energy and a unique incidence direction. Thus, 4BD may be used to precisely calibrate X-ray photon energies and beam positions. Based on the principles that the forbidden-reflection 4BD pattern, which is typically an X-shaped cross, can be generated by instant imaging using the divergent beam from a point source without rocking the crystal, a detailed real-time high-resolution beam (and source) position monitoring scheme is illustrated for monitoring two-dimensional beam positions and directions of modern synchrotron light sources, X-ray free-electron lasers and nano-focused X-ray sources.

## Abstract 2

A novel scheme based on four-beam diffraction is illustrated for accurate calibration of X-ray photon energies and real-time monitoring of two-dimensional beam positions of modern synchrotron light sources.

## 1. Introduction

X-ray diffraction from crystals is generally based on Bragg’s law of two-beam diffraction (2BD), 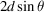 = λ, where θ is the Bragg angle, λ is the X-ray wavelength, and *d* is the spacing of the diffracting lattice planes. If λ can continuously vary, Bragg diffraction may occur at any angle 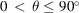 with no special points (DuMond, 1937 ▸). Without references, therefore, it is challenging to use Bragg diffraction to accurately calibrate/measure X-ray wavelengths (photon energies) or beam directions since it is usually difficult to measure θ with very high precision. As an alternative, X-ray absorption edges of elements have been widely used for wavelength calibration, particularly for double-crystal monochromators (DCMs) of synchrotron beamlines. For elements with sharp absorption features, the absorption method may give high energy precision (at the eV level or better) (Kraft *et al.*, 1996 ▸). In some unfavarouble cases, however, the absorption edges can be very wide (up to a few tens of electronvolts) (Keski-Rahkonen & Krause, 1974 ▸) without clear sharp features. This may make it difficult to achieve adequate precision so that one has to seek other alternatives.

The other disadvantage of Bragg diffraction is that it is only very sensitive to the incidence angle θ but insensitive to the lateral φ angle along the direction perpendicular to the *plane of incidence* (the plane containing both the principal incident wavevector and the diffraction vector). In other words, Bragg’s law of 2BD is mainly a one-dimensional (1D) equation that is unable to generate two-dimensional (2D) diffraction patterns required for precise determination of the 2D position and direction of an X-ray beam in space.

To overcome these problems, it has been proposed to use multiple-beam diffraction to calibrate photon energies (*e.g.* Arthur, 1989 ▸; Hagelstein *et al.*, 1992 ▸). However, these methods as well as many other existing multiple-beam diffraction methods involve wide-range Φ-scans that have extremely low resolution because the lateral beam divergence is generally much larger than the divergence along the θ angle for conventional X-ray sources. The fourth-generation synchrotrons (*e.g.* Pacchioni, 2019 ▸) and X-ray free-electron lasers (XFELs) (*e.g.* Emma *et al.*, 2010 ▸) with small 2D source sizes that can produce X-ray beams with high 2D collimation are expected to remove this obstacle. Motivated by the phase-space (1D) beam position monitor system based on element *K*-edge absorption (Samadi *et al.*, 2015 ▸, 2019*a* ▸,*b* ▸), here we propose a new scheme that can accurately monitor 2D source positions and directions and at the same time can calibrate the wavelengths of the X-ray beams. This scheme is based on forbidden-reflection four-beam diffraction (4BD) from crystals with cubic structures, which has to be activated by a unique photon energy and a unique beam direction. For a point source, the 4BD pattern is an X-shaped cross that can be generated by imaging without rocking the crystal. Therefore, this unique property may be used for real-time monitoring of beam positions. The 4BD method will be particularly suited for diagonstic applications of modern synchrotron light sources, XFELs, and nano-focused beams that have small 2D source sizes.

## 2. Four-beam diffraction patterns and properties

**Figure 1:**
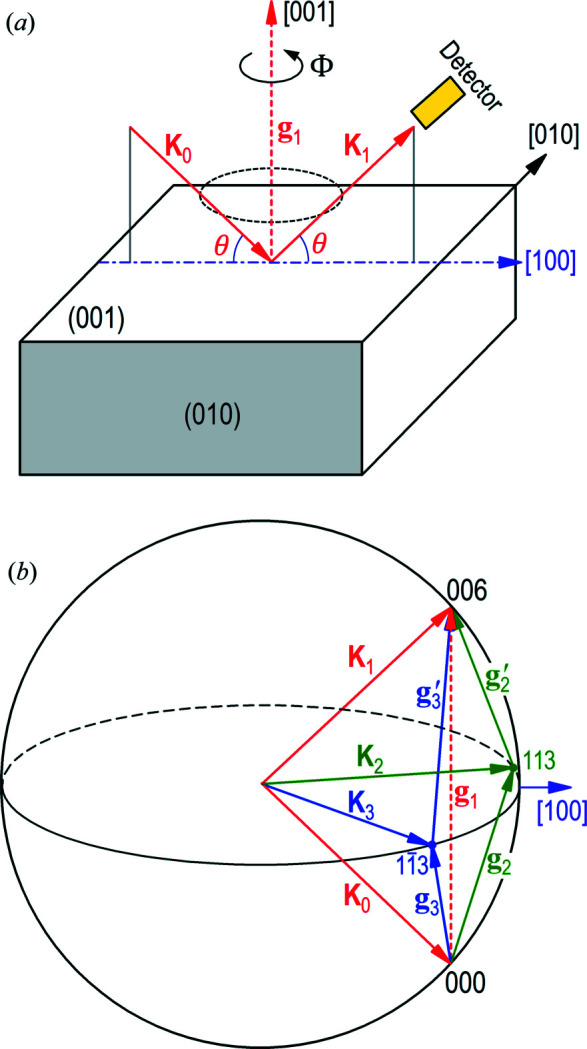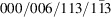 4BD configuration. (*a*) Diffraction geometry of the 006 main reflection in real space with the plane of incidence parallel to (010). **K**_0_ is the incidence wavevector, and **K**_1_ is the 006 reflection wavevector. The dashed circle indicates the diffraction cone of **g**_1_ formed by rotation of **K**_0_ and **K**_1_ around **g**_1_. (*b*) The Ewald sphere in reciprocal space. The four reciprocal lattice points 000, 006, 113 and 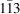 are all on the Ewald sphere. **g**_1_ = 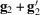 = 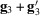 .

To demonstrate the concept, we study 4BD using silicon 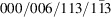 reflections^1^ with the lattice constant *a*_0_ = 5.431 Å. For a synchrotron X-ray source, this diffraction process can be activated by restricting the incident beam in the plane parallel to the (010) lattice plane, as shown in Fig. 1 ▸(*a*). Then one changes the incidence angle θ while keeping the incidence wavelength λ to satisfy the Bragg condition of the **g**_1_ = *h*_1_ *k*_1_ *l*_1_ = 006 reflection, 

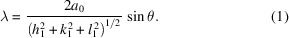

For specific wavelengths, some reciprocal lattice points may also fall on the Ewald sphere of reflection 006. Under this condition, multiple-beam diffraction occurs (Chang, 2004 ▸; Colella, 1974 ▸). For example, if the reciprocal lattice point *h*_2_ *k*_2_ *l*_2_ = 113 is on the Ewald sphere [Fig. 1 ▸(*b*)], it is obvious that the angle between its diffraction vector **g**_2_ = *h*_2_ *k*_2_ *l*_2_ = 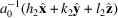 and the incident wavevector 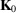 = 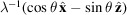 is π/2 + θ_113_, *i.e.*


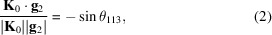

where θ_113_ is the Bragg angle of reflection 113 satisfying

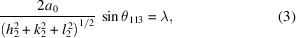

and 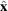 , 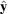 , 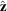 are unit vectors along the [100], [010], [001] directions, respectively. Then one can obtain 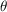 = 40.6013° from equations (1)–(3). We denote this angle by θ_4B_. The corresponding photon energy is *E*_4B_ = 10.5236 keV. By symmetry, the 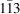 reciprocal lattice point is also located on the Ewald sphere when **K**_0_ lies in the (010) plane. Therefore, under these conditions, Fig. 1 ▸ represents a 4BD configuration involving reflections 000, 006, 113 and 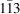 (Huang *et al.*, 2014*a* ▸,*b* ▸), and we call **g**_1_ = 006 the *main* reflection.

Here we are only interested in the diffraction intensity of the main reflection 006 along the **K**_1_ direction in Fig. 1 ▸. This implies that the experimental 4BD setup will be almost identical to that of 2BD, where one sets the detector to collect only the main reflection intensity. The intensities of the other two reflections **g**_2_ = 113 and **g**_3_ = 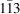 are usually of no importance. The only difference is that 4BD requires a high-resolution (∼1 µrad) rotation stage for accurate control of the azimuthal angle Φ in Fig. 1 ▸, while in 2BD the diffraction pattern is invariant with Φ for symmetric reflections.

Another interesting property of multiple-beam diffraction is that some of the involved reflections can be forbidden reflections. For the 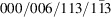 4BD case, the main reflection 006 of silicon is forbidden, but it can still produce strong diffraction along the **K**_1_ direction in Fig. 1 ▸. The underlying mechanism is that the diffraction intensity along **K**_1_ comes from two *detour reflection channels* in Fig. 1 ▸(*b*). In one channel, the incident beam is first diffracted by **g**_2_, *i.e.* **K**_0_ + **g**_2_ = **K**_2_. Then this diffracted wave is further diffracted by 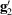 = **g**_1_ − **g**_2_ = 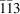 to produce a diffracted wave with wavevector **K**_1_, *i.e.* 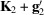 = **K**_1_. Similarly, the other channel consists of the two steps **K**_0_ + **g**_3_ = **K**_3_ and 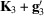 = **K**_1_, where 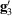 = 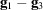 = 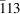 (Huang *et al.*, 2014*a* ▸,*b* ▸). Since the direct diffraction channel **K**_0_ + **g**_1_ = **K**_1_ is forbidden, the diffraction pattern detected along the **K**_1_ direction is a pure multiple-beam diffraction effect without the contribution of direct 2BD. Note that multiple-beam diffraction with a forbidden main reflection has been experimentally verified and it has many applications (*e.g.* Lang *et al.*, 2013 ▸).

**Figure 2:**
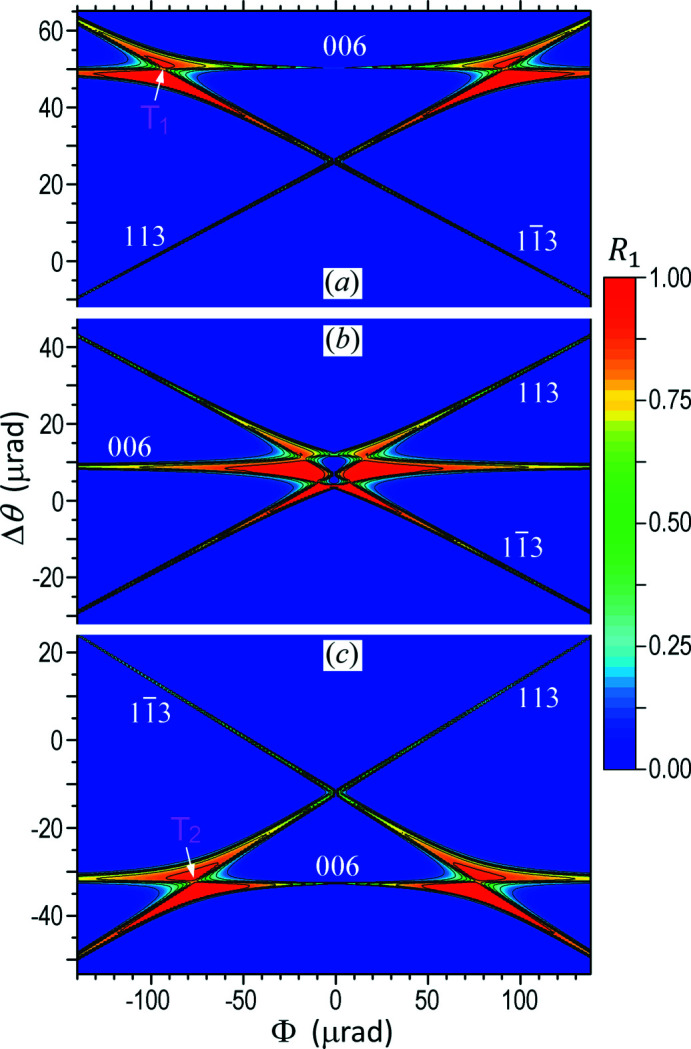
Computed 2D ‘rocking curve’ of the 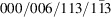 4BD setup in Fig. 1 ▸ showing the reflectivity *R*_1_ of the main reflection **g**_1_ = 006 as a function of the incidence angle (Φ, Δθ) and the photon energy *E*. The indices of each line (diffraction cone) indicate the corresponding Bragg reflection. σ-polarization, *i.e.* the electric field of the incident wave, is parallel to [010] in Fig. 1(*a*). Semi-infinite Si crystal. Δθ = 0 always corresponds to θ_4B_ = 40.6013°. (*a*) *E* = *E*_4B_ − 0.5 eV. (*b*) *E* = *E*_4B_. (*c*) *E* = *E*_4B_ + 0.5 eV.

X-ray multiple-beam diffraction from perfect crystals can be rigorously computed by the Fourier coupled-wave diffraction theory (FCWDT) (Huang *et al.*, 2013 ▸; Tang *et al.*, 2021 ▸), which is based on similar principles as the method developed by Stetsko & Chang (1997 ▸). But, the FCWDT is more efficient and accurate. Using the FCWDT, we have calculated the 2D distribution of the reflectivity *R*_1_ along the **K**_1_ direction for the 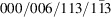 4BD configuration, as shown in Fig. 2 ▸. Here we always set the main reflection to be symmetric, *i.e.* **g**_1_ is strictly perpendicular to the crystal surface. We allow the incident beam to slightly deviate from the (010) plane by an azimuthal angle Φ. Then the incident wavevector is **K**_0_ = 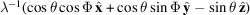 in Fig. 1 ▸. The reflectivity only depends on **K**_0_, *i.e.* *R*_1_ is a function of Φ, θ, and the photon energy *E*. Fig. 2 ▸(*b*) shows the calculated *R*_1_ distribution as a function of Φ and θ at the exact Bragg energy *E*_4B_ = 10.5236 keV. Δθ is the relative deviation of the incidence angle from the geometrical Bragg angle θ_4B_ = 40.6013°. This map represents a 2D ‘rocking curve’ for an incident plane wave.

Note that for 2BD the Bragg condition is maintained when the incident and diffracted beams are rotated around the diffraction vector. We call the cone formed by such rotation the *diffraction cone*. For example, azimuthal rotation of **K**_0_ and **K**_1_ along **g**_1_ in Fig. 1 ▸(*a*) forms a cone that is the diffraction cone of **g**_1_. The diffraction pattern in Fig. 2 ▸(*b*) mainly consists of three lines, which correspond to the diffraction cones of the three reflections 006, 113, and 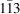 , respectively, in the Φ–θ coordinate system. For example, the horizontal line corresponds to the 006 reflection, of which the Bragg condition is independent of Φ. All three lines intersect in the central region that is the 4BD region. In this region, the three lines have significant distortions, indicating that the three reflections have strong interactions. Here also note that the detour reflection can be very strong, with reflectivity close to 100% in some regions although the direct 006 reflection is forbidden.

Fig. 2 ▸(*a*) is the diffraction pattern when the photon energy *E* deviates from *E*_4B_ by Δ*E* = −0.5 eV. Consequently, the Bragg angle of the 006 reflection increases following equation (1), as indicated by the upward shift of the 006 diffraction line (cone). The 113 and 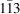 diffraction cones also change with *E* according to their respective Bragg equations, for instance, equation (3) for reflection 113. The resulting effect is that the three lines no longer intersect at the same point, indicating that the 4BD condition can no longer be satisfied when the incident energy deviates from *E*_4B_. Instead, each pair of lines intersect at a distinct point that corresponds to a three-beam diffraction (3BD) process (Renninger, 1937 ▸; Lang *et al.*, 2013 ▸). The top-left (T_1_) and top-right intersections in Fig. 2 ▸(*a*) correspond to the 000/006/113 and 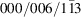 3BD processes, respectively, while the intersection in the central area corresponds to 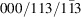 3BD. Fig. 2 ▸(*c*) is the diffraction pattern for *E* = *E*_4B_ + 0.5 eV, and it is nearly a mirror image of Fig. 2 ▸(*a*) with respect to the central 006 diffraction line Δθ = 8 µrad in Fig. 2 ▸(*b*). Here the slight shift of the central line from Δθ = 0 in Fig. 2 ▸(*b*) is caused by the slight refraction effect of X-rays.

The most important phenomenon revealed from Fig. 2 ▸ is that 4BD can only occur along a unique incidence direction and for a unique photon energy. For the current case, the unique energy is *E*_4B_ = 10.5236 keV, which is determined only by the lattice constant *a*_0_. The unique incidence direction corresponds to Δθ = 8 µrad (relative to θ_4B_ = 40.6013°) and Φ = 0 [relative to the (010) lattice plane].

**Figure 3:**
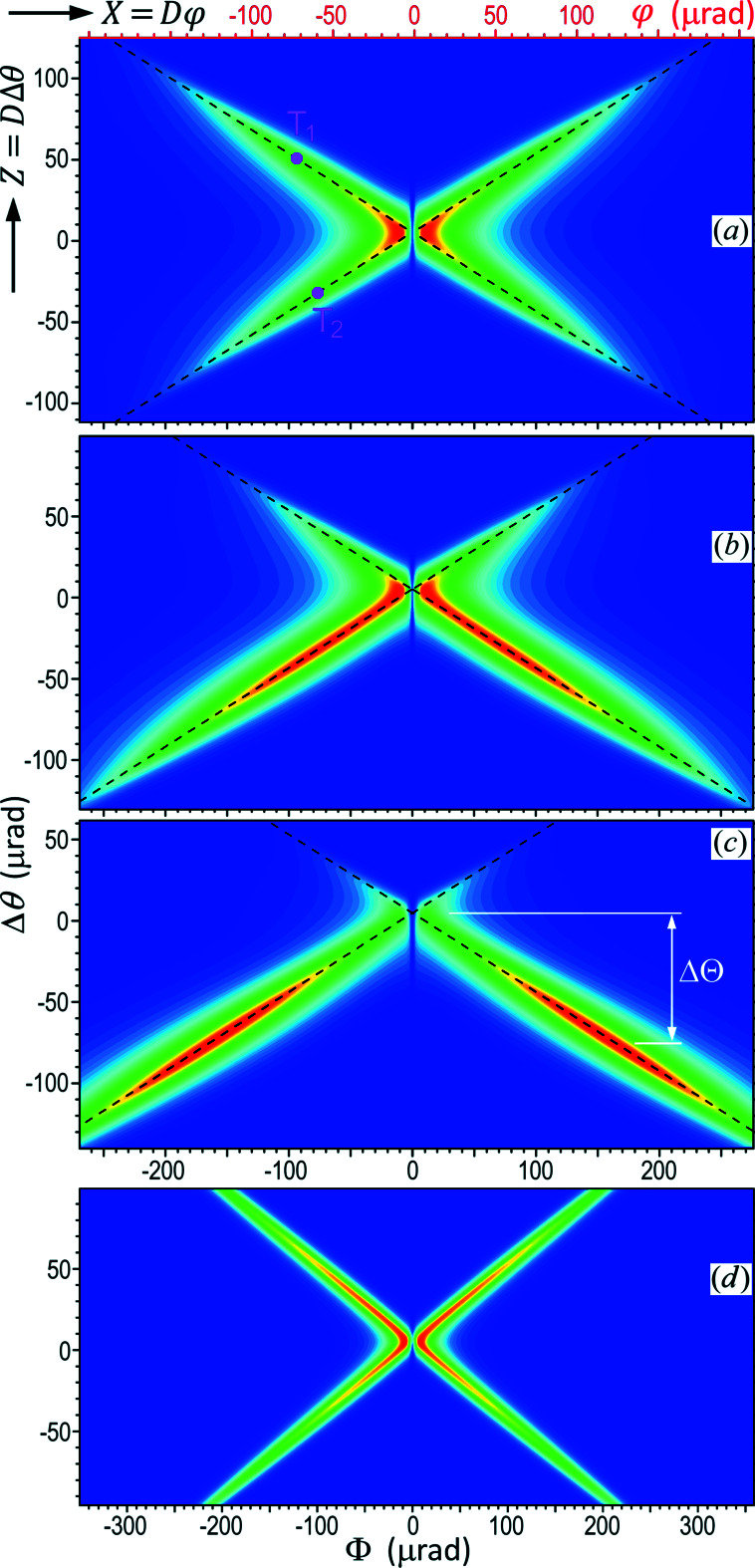
(*a*–*c*) Integrated 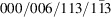 4BD patterns for a Gaussian incidence spectrum (Δ*E*_BW_ = 1.5 eV). The intensities are normalized intensities of the main reflection **g**_1_ = 006. The patterns can be generated with a parallel incident beam by 2D rocking of the crystal, or with a divergent beam from a point source as a one-shot image in the *XZ* coordinate system. Δθ = 0 corresponds to θ_4B_ = 40.6013°. (*a*) Central photon energy of the Gaussian spectrum *E*_c_ = *E*_4B_ (= 10.5236 keV). (*b*) *E*_c_ = *E*_4B_ + 0.5 eV. (*c*) *E*_c_ = *E*_4B_ + 1 eV. (*d*) Integrated 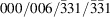 4BD pattern (*E*_c_ = *E*_4B_ = 8.44807 keV). The color scale is the same as in Fig. 2 ▸.

Fig. 2 ▸ shows that the plane-wave 4BD pattern is very sensitive to photon energy variation. For a real X-ray beam, the spectrum always has a finite energy bandwidth. Here we consider a synchrotron radiation beam monochromated by an upstream Si (111) DCM. The spectral bandwidth of the beam after the DCM is Δ*E*_BW_ ≃ 1.5 eV at 10.5 keV, and we assume that the intensity has a Gaussian distribution in terms of *E*. Based on such an incident beam, we have computed the plane wave diffraction patterns (as in Fig. 2 ▸) for different energies within the DCM bandwidth and overlapped them together to form the integrated diffraction pattern in Fig. 3 ▸(*a*). Here the center of the Gaussian spectrum is *E*_4B_. The pattern is an X-shaped cross consisting of two oblique bands. Here, note that the central lines of the two bands (dashed lines) are not the diffraction cones in Fig. 2 ▸. The diffraction cones in Fig. 2 ▸ are for monochromatic beams, and their cone apertures (2θ_B_) vary with the photon energy *E* according to their respective Bragg equations, as indicated by the displacements of the lines with *E* in Fig. 2 ▸. In contrast, the two oblique dashed lines in Figs. 3 ▸(*a*)–3(*c*) have fixed positions although the diffraction intensity distribution on the lines changes with varying *E*. The other obvious difference is that the oblique lines in Figs. 2 ▸ and 3 ▸(*a*)–3(*c*) have different slopes.

Detailed analyses show that the two dashed lines in Figs. 3 ▸(*a*)–3(*c*) are the loci of the monochromatic 000/006/113 and 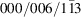 3BD centers for different photon energies. For examples, the line intersection centers T_1_ and T_2_ in Fig. 2 ▸ are indeed located on the two dashed lines in Fig. 3 ▸(*a*). Such intersections move in the Φ–θ coordinate system with varying *E*, thus forming the two 3BD lines that can extend far away if the energy and angular ranges are wide enough. Based on this conclusion, one can use the Bragg equations and equation (2) to easily calculate the slopes and positions of dashed lines in the Φ–θ space. On the other hand, this indicates that 3BD can continuously occur for any wavelengths allowed by the diffraction geometry, similar to 2BD. Hence, 3BD usually cannot be used for energy calibration. However, the intersection of the two dashed line in Fig. 3 ▸, here called the cross center (CC), is the 4BD center that corresponds to a unique photon energy and a unique incidence direction even for polychromatic incident beams. This can be seen from Figs. 3 ▸(*b*) and 3 ▸(*c*) that when the central energy of the incident Gaussian spectrum deviates from *E*_4B_ the strong diffraction regions move away from the fixed-position CC to cover different regions of the X-shaped cross. The strong diffraction centers are always on the two fixed dashed lines. Here it can be verified that the shift of the strong diffraction center from the CC, ΔΘ, along the Δθ axis [see Fig. 3 ▸(*c*)] and the shift of the spectral center, Δ*E*, satisfy the differential Bragg law of the 006 reflection,

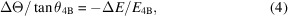

around θ_4B_. Hence, different points with different angles on the dashed lines correspond to different energies. Only those points with the corresponding energies falling within the incident spectrum can have strong diffraction when the incidence direction is correct. If *E* is away from *E*_4B_, the CC may be completely out of diffraction. In such circumstances, one can still extrapolate the measured dashed line segments [*e.g.* in the lower half of Fig. 3 ▸(*c*)] to derive the CC position. This is very useful for the initial search and alignment of the 4BD crystal orientation and also for monitoring X-ray beams with large variations.

Therefore, a polychromatic incident beam can produce a unique X-shaped diffraction pattern in the vicinity of the 4BD region. In particular, the spectral bandwidth and the divergence of the incident beam only affect the extent of the diffraction pattern (in the Φ–θ space) and its intensity distribution without affecting the position of the CC. But, note that a white beam will not work here since it will activate strong harmonic reflections, such as 004 and 008, that will significantly reduce (if not destroy) the visibility of the 006 reflection. This is the reason why we have considered in the above a synchrotron beam monochromated by a Si (111) DCM.

For comparison, Fig. 3 ▸(*d*) shows the integrated diffraction pattern for another example of 4BD, 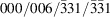 with θ_4B_ = 54.1623° and *E*_4B_ = 8.44807 keV, where the bandwidth of the incident beam is Δ*E*_BW_ = 1.2 eV, which is the bandwidth of a Si (111) DCM for this energy. Here the cross is sharper than those in Figs. 3 ▸(*a*)–3(*c*). For higher energies, the 4BD pattern can be further sharper. Note that the sharpness of the cross directly affects the precision of BPM and energy calibation.

Thus, we have illustrated that practical 4BD experiments with finite-bandwidth incident beams can be used to calibrate X-ray photon energies and measure the directions of X-ray beams based on the fact that the CC in Fig. 3 ▸ corresponds to a unique energy and a unique incidence direction. Although the polychromatic diffraction patterns are not as sharp as the plane-wave diffraction patterns, it is evident that the energy resolution can reach the sub-eV level from the comparison of Figs. 3 ▸(*a*)–3(*c*). Meanwhile, the angular resolution can reach the µrad level. The beam direction determined by 4BD is a complete 2D orientation (with the same high resolution along both the θ and Φ directions), which is a remarkable advantage over 2BD that may be sensitive only to the 1D directional change Δθ.

## 3. Energy calibration and beam position monitoring of point sources

In principle, the diffraction patterns in Fig. 3 ▸ can be realized by 2D rocking of the crystal near the 4BD conditions using a parallel polychromatic incident beam, but such a 2D scanning process will be time-consuming. Meanwhile, X-ray beams with 2D high collimation are very difficult to obtain and most X-ray beams have larger lateral (φ) divergence as aforementioned. In the following, we will focus on the principles of an imaging method that uses a naturally divergent beam from a point source to form a one-shot image of the 4BD pattern.

**Figure 4:**
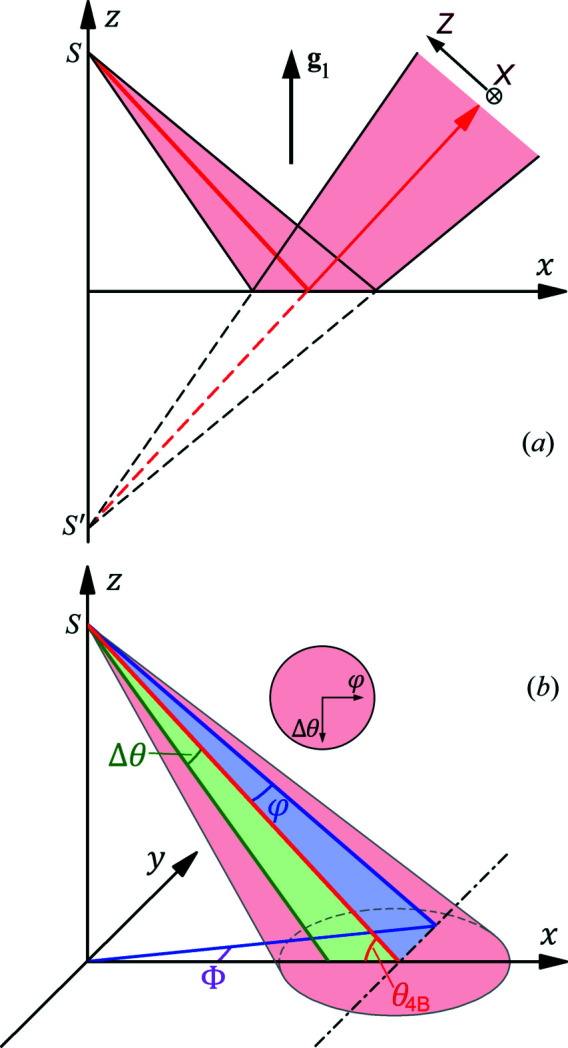
(*a*) Mirror reflection law of symmetric Bragg reflection. The virtual source *S*′ is exactly the mirror image of the real point source *S* with respect to the crystal surface (*z* = 0). (*b*) Relationship between the lateral divergence angle φ and the azimuthal angle Φ on the crystal surface (*z* = 0). The inset indicates the cross section of the divergent beam viewed along the direction opposite to the beam direction.

First, note that symmetric Bragg reflections always follow the flat mirror reflection (specular reflection) law for ray directions, which results from the conservation of the tangential momenta, **K**_0||_ + **g**_*n*||_ = **K**_*n*||_. Here || denotes the tangential component of the corresponding vector, *i.e.* the projection of the vector onto the crystal surface. This equation is valid for any Bragg reflection (Huang *et al.*, 2013 ▸). For the symmetric main reflection **g**_1_ in Fig. 1 ▸, **g**_1||_ = 0 leads to **K**_1||_ = **K**_0||_. Consequently, |**K**_1_| = |**K**_0_| gives *K*_1*z*_ = −*K*_0*z*_. For symmetric Bragg reflections (of two- or multiple-beam diffraction), this mirror reflection law is always valid *for any incidence directions and any wavelengths*. [Asymmetric reflections are dispersive and do not have this property (Huang *et al.*, 2012 ▸).] In the case of a point source, therefore, the symmetric Bragg reflection gives rise to a mirror-image virtual source *S*′, as shown in Fig. 4 ▸(*a*). Viewed from *S*′, each ray will not change its direction at all during the Bragg reflection. The only difference of the Bragg reflection is that only X-rays falling within a narrow spectral bandwidth and in a very small angular range will be strongly reflected, while mirror reflection of light is usually achromatic for any incidence direction. Similarly, a DCM consisting of two identical symmetric reflections also exactly preserves the direction of each ray, where the second reflection exactly offsets the deflection angle of the first one. The net effect is that the DCM only causes a beam displacement that has no effect on the subsequent imaging process.

Second, the 2D orientation of a ray emitted from a point source can be described by two small angles along two orthogonal directions Δθ and φ with respect to the central axis of the beam, as shown in Fig. 4 ▸(*b*). For synchrotron beamlines, these two directions usually correspond to the vertical and horizontal divergence directions, respectively. For the vertical Bragg reflection, the vertical divergent angle Δθ is the same as the variation of the incidence angle θ on the crystal surface in Fig. 4 ▸(*b*). In addition to θ, we have also used the azimuthal angle Φ above in Figs. 2 ▸ and 3 ▸ to describe the incidence wavevector **K**_0_ = 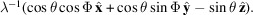 From Fig. 4 ▸(*b*) it is apparent that the lateral (horizontal) divergence angle φ is proportional to the azimuthal angle Φ we used in the above calculations by a geometrical factor 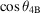 , *i.e.* φ = 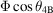 for small φ. For the current case, 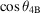 = 0.76.

Hence, a point source naturally provides X-rays along all the various directions around the central axis of the beam. When the divergent beam is incident on the crystal, each point on the illuminated surface corresponds to a unique incidence direction (φ, θ). Consequently, the point source may generate the 4BD patterns in Fig. 3 ▸ as one-shot images without the necessity of 2D angular scans of the crystal. In Fig. 3 ▸, we have set the Δθ and φ (top) axes to have the same scale. Then the one-shot images will have exactly the same shapes as the simulated images if the imaging device (usually a charge-coupled device, CCD) is set perpendicular to the diffracted beam. As shown in Fig. 4 ▸(*a*), if we establish the *XZ* coordinate system on the CCD, the positions on the CCD are related to the angular directions by *X* = *D*φ and *Z* = *D*Δθ, where *D* is the distance between the point source and the CCD. If we assume *D* = 50 m for a synchrotron beamline, the image size in Fig. 3 ▸(*a*) will be 2.04 cm × 1.18 cm, which is close to the fields of view of some typical X-ray CCDs. If the beam divergence is smaller than that in Fig. 3 ▸, one can adjust the DCM and the crystal to image only the central part of the diffraction pattern.

The above principles show that a point source can be used to perform real-time imaging of the 2D 4BD pattern without angular scans of the crystal. In experiments, the initial alignment may still need angular scans. One can first set the DCM energy roughly at *E*_4B_ and set the Bragg angle of **g**_1_ = 006 roughly at θ_4B_. Then fine alignment of the crystal to the exact 4BD conditions may require several test Φ scans at different θ angles. Once we find two symmetric 3BD points on the two oblique dashed lines in Fig. 3 ▸, the two lines can be immediately determined in the Φ–θ space based on their (calculated) slopes, from which the CC position can be derived. Afterwards, one sets the crystal orientation to the derived CC position and makes a DCM energy scan to maximize the 4BD intensity. Under this condition, the DCM energy (center of the spectrum) is calibrated to *E*_4B_ with an accuracy better than 0.5 eV. Meanwhile, the crystal orientation is also precisely determined.^2^

In practice, the finite source size may blur the images in Fig. 3 ▸ in the imaging method. However, for modern low-emittance synchrotron light sources or XFELs with source sizes Δ*s* < 50 µm and beamline lengths *D* > 10 m, the source size effect Δ*s*/*D* < 5 µrad is very small, which can be further reduced or eliminated by image processing and data fitting. For the current cases, from data fitting the energy calibration precision may reach up to 0.1 eV. In a reversed way, data fitting of the image blurring can also give information about the source size.

**Figure 5:**
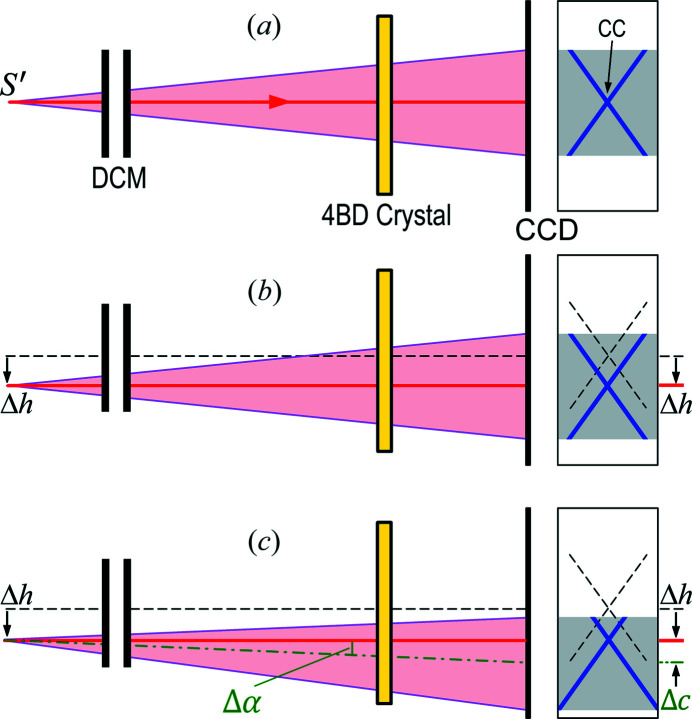
Schematic of the BPM using 4BD of a point source. The left-hand side shows side views. The right-hand side shows the CCD viewed along the beam direction (**K**_1_ in Fig. 1 ▸). The beam displacement caused by the DCM is not shown, which does not affect the BPM. Since the deflection angle (2θ_4B_) of the 4BD crystal is omitted, the source *S*′ is the virtual source in Fig. 4 ▸(*a*). For simplicity the 2D variations of the source, the beam and the image are indicated by 1D shifts. (*a*) Initial 4BD configuration. (*b*) *S*′ is displaced by Δ*h* without beam direction changes. (*c*) *S*′ is displaced by Δ*h* together with a beam direction change Δα, which makes the 4BD pattern asymmetric as the image center is shifted by Δ*c* = *D*Δα from the CC.

In addition to wavelength calibration, the most potential application of the 4BD imaging method is for beam position monitoring (BPM) of modern synchrotron light sources and XFELs, and the principles can be understood from Fig. 5 ▸. In Fig. 5 ▸(*a*), we assume that the DCM and the crystal are aligned to the 4BD conditions to produce a symmetric 4BD pattern on the CCD detector. For simplicity of description, we further assume that the thick red line connecting the (virtual) source and the CC of the 4BD pattern is exactly horizontal. After the initial alignment, we assume that the DCM and the crystal are strictly static. Now in Fig. 5 ▸(*b*) if the source is displaced in the transverse plane by a distance Δ*h*, the horizontal red line containing the source will also be displaced by Δ*h*. As described above, the CC of the 4BD pattern always corresponds to an exact unique incidence direction, which is the horizontal direction here. This indicates that the CC on the CCD will also be displaced by the same Δ*h*. Hence, the CC on the CCD will move synchronously with the source along the same direction. Therefore, monitoring the movement of the CC on the CCD is equivalent to real-time monitoring of the beam/source position. If the resolution of the CCD is on the micrometre level, it is expected that this BPM method can achieve micrometre resolution. Data fitting may further improve the spatial resolution.

As shown by Samadi *et al.* (2015 ▸), not only the position of a synchrotron light source or XFEL may vary over time, but the beam direction (angle) can also change. Fig. 5 ▸(*c*) schematically shows that the X-ray beam has both a displacement Δ*h* and an orientation variation Δα. It is obvious that as long as the horizontal direction remains within the angular range of the divergent beam, the additional Δα does not affect the displacement of the CC, which is still Δ*h*. Under the condition that the beam divergence is small enough, however, the angular variation of the beam will make the 4BD pattern asymmetric with the image center (which can be calculated by data fitting if necessary) shifted from the CC by a distance of Δ*c* = *D*Δα if the divergence of the beam is small enough. If the beam divergence is large, measurements of Δ*c* may not be very accurate. Meanwhile, the variation of the 4BD pattern from the symmetric shapes in Figs. 5 ▸(*a*) and 5 ▸(*b*) caused by beam direction changes may also depend on the spectrum (which can be numerically computed). However, all these factors do not affect the beam position variation Δ*h* if the beam direction variation remains reasonably small. (For large beam direction variation, the 4BD could be out of the angular range of the incident beam.) Obviously, the 4BD BPM scheme, in fact, monitors the true position of the X-ray source (electron beam source) and, compared with many conventional BPM methods, a unique property of this method is that the source position and variation revealed are completely independent of any slits (particularly beam-defining slits or devices) that may exist in the beamline.

For simplicity, the variations of the beam and the image in Fig. 5 ▸ are schematically indicated by the 1D movements of the images, but it is obvious that in experiments the image variation gives the 2D variations of the source position and beam directions since the 4BD pattern is a 2D cross. As mentioned above, this is a remarkable advantage over 2BD schemes that can only give 1D beam position variation.

Fig. 3 ▸ indicates that imaging a complete 4BD pattern (for *E* < 12 keV) requires beam divergence of several hundreds of µrad along both direction. Although bending-magnet beamlines can meet such requirements, the beam divergence of fourth-generation synchrotron undulators and XFELs may be only a few tens of µrad (for open beams without slits). However, note that here the BPM only depends on the position of the CC, which can be determined by a small central region around the CC without the necessity to image the entire 4BD pattern. For example, one can estimate that in Fig. 3 ▸(*d*), where the 4BD pattern has sharp lines, a realistic divergence range of about 20 µrad (V) × 40 µrad (H) in the central part may well determine the CC position. As mentioned above, the 4BD pattern can become much sharper at higher energies (>15 keV), which can further reduce the requirement of beam divergence if the BPM is chosen to work at these energies.

## 4. Discussion and conclusion

The above principles of 4BD and the corresponding point source imaging scheme are based on rigorous dynamical theory calculations. A practical implementation of the scheme at a synchrotron beamline for BPM will have two challenging requirements. The first is to maintain high stability of the DCM, particularly the stability of the relative orientation between the two DCM crystals. Variation of this relative orientation directly alters the virtual source position. It also changes the spectrum and beam direction, though these two factors are less critical than the virtual source variation that directly affects the precision and reliability of the BPM. On the other hand, by frequency analyses, it is also possible to decouple and identify the origin of beam motion (vibration) from either the source or the optics (Samadi *et al.*, 2019*c* ▸).

**Figure 6:**
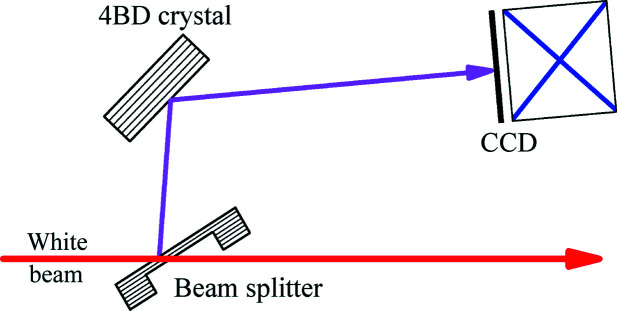
Replacement of the DCM with a thin-crystal window for a 4BD-based BPM with possibly low heat load and minimum thermal lattice distortion on the thin crystal. The thin crystal must be of symmetric Bragg reflection.

The second requirement is to reduce the heat load of the DCM so as to mimimize thermal lattice distortion (better than 10^−5^). Lattice distortion (similar to slope errors of mirrors) will significantly change the directions of the locally diffracted X-rays and thus change the virtual source size and position, which may significantly affect or even destroy the 4BD pattern. A possible way to mitigate the heat load is to use a thin-crystal window as a beam splitter (Osaka *et al.*, 2013 ▸) to diffract a weak beam for the 4BD imaging setup, as schematically shown in Fig. 6 ▸. This scheme has three major advantages. First, the head load of the thin crystal may be very small since the crystal thickness can be as thin as a few micrometres such that most of the photons will be transmitted without absorption. (Setting homogeneous absorbers/filters upstream of the thin crystal may reduce the long-wavelength heat load.) Second, the spectral bandwidth of the beam diffracted by the thin crystal can be wider than that diffracted by a thick crystal. The wide bandwidth can extend the angular range of the 4BD pattern in Fig. 3 ▸. Third, it is apparent that with the use of a thin-crystal splitter the 4BD-based BPM process will not require a dedicated beamline. Hence it may be implemented at any beamline with little effect on the main beamline.

Although we have focused on the 4BD case around 10 keV, there are, in fact, a large number of such 4BD configurations that cover various energies. For *E* < 20 keV, Table 1 ▸ lists some typical 4BD cases of silicon with a (001) surface and with the same (010) plane of incidence. Note that one can use a single silicon crystal with the (001) surface to realize all these 4BD configurations simply by setting the Bragg angle of the main reflection (**g**_1_ = 002 or 006) to the specific θ_4B_. If the crystal is rotated by 45° such that the plane of incidence becomes the (110) plane that also has mirror symmetry, we can obtain another set of 4BD configurations.

In fact, some of the special 4BD configurations in Table 1 ▸ involve six or eight reflections (or more). For such six- and eight-beam diffraction geometry, the diffraction patterns consist of three and four lines, respectively, instead of only two lines in Fig. 3 ▸ for 4BD. All these lines intersect at the same point that is the center of the diffraction pattern as in Fig. 3 ▸(*a*). Therefore, the diffraction mechanisms of six- or eight-beam diffraction is almost the same as 4BD for energy calibration and for BPM. As mentioned above, the slopes and positions of all the inclined lines can be calculated by a simple computer program using simple geometry without the complicated dynamical theory calculations.

The multiple-beam diffraction can also be arranged in the transmission geometry, which may generate additional unique features owing to the transmission-diffraction properties, such as the Borrmann effect (Okitsu *et al.*, 2003 ▸). For photon energies below 10 keV, however, the transmission geometry requires thin crystals that can be easily strained, which may require strain-mitigation efforts.

The 4BD principles are also valid for the case where **g**_1_ is not forbidden. In this circumstance, however, the 4BD pattern has a strong and wide vertical column that is mainly the direct two-beam diffraction intensity of **g**_1_ (nearly independent of Φ in the regions away from the CC). This column will greatly reduce or hide the sharp contrast features around the CC in Fig. 3 ▸.

In summary, we have used rigorous dynamical-theory calculations to demonstrate that the forbidden-reflection 4BD pattern, which is typically an X-shaped cross, can be activated only by a unique photon energy and a unique incidence direction. The 4BD geometry can be used to calibrate photon energies and X-ray beam positions and directions. Based on the principles that the 4BD pattern can be generated by direct imaging using the divergent beam from a point source without crystal rocking, we illustrated a high-resolution beam position monitoring scheme for real-time monitoring or ultrafast imaging of the sources of synchrotron beamlines and nano-focused beams. Although other BPMs, particularly blade-type BPMs, that can differentiate both the position (with µm resolution) and angle (down to ∼1 µrad) of the beam have already been developed and widely used (*e.g.* Huang *et al.*, 2019 ▸), the new scheme we presented here provides an alternative method that is based on completely different mechanisms and may have unique or complementary advantages. We admit that the instant imaging of the 4BD pattern may be challenging for X-ray beams with extremely small divergence (*e.g.* fourth-generation light sources with divergence ∼10 µrad), which requires testing (preferably at higher photon energies with sharper 4BD patterns) in the future. However, we are confident that our scheme should work perfectly for bending-magnet or wiggler beamlines. In our following work, we will test this scheme at beamline 1-BM of the Advanced Photon Source, but note that the work by Samadi *et al.* (2015 ▸) has already demonstrated the feasibility for the 1D case.

## Figures and Tables

**Table 1:** Typical 4BD reflections from the silicon (001) surface for *E* < 20 keV

4BD reflections	θ_4B_ (°)	*E*_4B_ (keV)
000/002/\bar 111/\bar 1\bar 11	63.4349	2.55235
000/002/\bar 311/\bar 3\bar 11	33.6901	4.11554
000/002/\bar 31\bar 1/\bar 3\bar 1\bar 1	24.7751	5.44767
000/002/\bar 113/\bar 1\bar 13	21.8014	6.14687
000/006/\bar 333/\bar 3\bar 33	63.4349	7.65705
000/006/\bar 331/\bar 3\bar 31	54.1623	8.44807
000/006/\bar 531/\bar 5\bar 31	45.9710	9.52543
000/006/113/1\bar 13	40.6013	10.5236
000/006/\bar 553/\bar 5\bar 53	36.1932 ▸	11.5979
000/006/\bar 753/\bar 7\bar 53	32.8687	12.6193
000/006/\bar 755/\bar 7\bar 55	31.3287	13.1719
000/006/\bar 953/\bar 9\bar 53	29.1047	14.0801
000/006/\bar 757/\bar 7\bar 57	27.4076	14.8782
000/006/\bar 957/\bar 9\bar 57	25.5420	15.8838
000/006/\bar 775/\bar 7\bar 75	24.3045	16.6397
000/006/\bar 759/\bar 7\bar 59	22.5796	17.8367
000/006/\bar 977/\bar 9\bar 77	21.5124	18.6764
000/006/\bar 577/\bar 5\bar 77	20.3231	19.7189

## Footnotes

1 The  case is, in fact, a six-beam diffraction configuration also exciting reflections  and  (Tang *et al.*, 2021 ▸), but these two reflections are very weak and are ignored here for simplicity of illustration.

2 This indicates that for angular-scan-based energy calibration, a time-consuming full 2D Φ–θ is not always necessary although such a full scan can be used to verify the calibration.

## References

[bb1] Arthur, J. (1989). *Rev. Sci. Instrum.* **60**, 2062–2063.

[bb2] Chang, S.-L. (2004). *X-ray Multiple-Wave Diffraction: Theory and Applications*, Vol. 143 of *Solid State Sciences Series*. Berlin: Springer-Verlag.

[bb3] Colella, R. (1974). *Acta Cryst.* A**30**, 413–423.

[bb4] DuMond, J. (1937). *Phys. Rev.* **52**, 872–883.

[bb5] Emma, P. *et al.* (2010). *Nat. Photon.* **4**, 641–647.

[bb6] Hagelstein, M., Cunis, S., Frahm, R. & Rabe, P. (1992). *Rev. Sci. Instrum.* **63**, 911–913.

[bb7] Huang, C.-H., Wu, C.-Y., Chiu, P.-C., Cheng, Y.-S., Liao, C.-Y., Hu, K.-H. & Hsu, K.-T. (2019). *AIP Conf. Proc.* **2054**, 060053.

[bb8] Huang, X.-R., Jia, Q., Wieczorek, M. & Assoufid, L. (2014*a*). *J. Appl. Cryst.* **47**, 1716–1721.

[bb9] Huang, X. R., Macrander, A. T., Honnicke, M. G., Cai, Y. Q. & Fernandez, P. (2012). *J. Appl. Cryst.* **45**, 255–262.

[bb10] Huang, X.-R., Peng, R.-W., Gog, T., Siddons, D. P. & Assoufid, L. (2014*b*). *Appl. Phys. Lett.* **105**, 181903.

[bb11] Huang, X.-R., Peng, R.-W., Hönnicke, M. G. & Gog, T. (2013). *Phys. Rev. A*, **87**, 063828.

[bb12] Keski-Rahkonen, O. & Krause, M. O. (1974). *At. Data Nucl. Data Tables*, **14**, 139–146.

[bb13] Kraft, S., Stümpel, J., Becker, P. & Kuetgens, U. (1996). *Rev. Sci. Instrum.* **67**, 681–687.

[bb14] Lang, R., de Menezes, A. S., dos Santos, A. O., Reboh, S., Meneses, E. A., Amaral, L. & Cardoso, L. (2013). *J. Appl. Cryst.* **46**, 1796–1804.

[bb15] Okitsu, K., Imai, Y., Ueji, Y. & Yoda, Y. (2003). *Acta Cryst.* A**59**, 311–316. 10.1107/s0108767303009036 12832809

[bb16] Osaka, T., Yabashi, M., Sano, Y., Tono, K., Inubushi, Y., Sato, T., Matsuyama, S., Ishikawa, T. & Yamauchi, K. (2013). *Opt. Express*, **21**, 2823–2831. 10.1364/OE.21.002823 23481739

[bb17] Pacchioni, G. (2019). *Nat. Rev. Phys.* **1**, 100–101.

[bb18] Renninger, M. (1937). *Z. Phys.* **106**, 141–176.

[bb19] Samadi, N., Bassey, B., Martinson, M., Belev, G., Dallin, L., de Jong, M. & Chapman, D. (2015). *J. Synchrotron Rad.* **22**, 946–955. 10.1107/S1600577515007390 PMC4489536 26134798

[bb20] Samadi, N., Shi, X. & Chapman, D. (2019*b*). *J. Synchrotron Rad.* **26**, 1863–1871. 10.1107/S1600577519010658 31721728

[bb21] Samadi, N., Shi, X., Dallin, L. & Chapman, D. (2019*a*). *J. Synchrotron Rad.* **26**, 1213–1219. 10.1107/S1600577519005423 PMC6613114 31274446

[bb22] Samadi, N., Shi, X., Dallin, L. & Chapman, D. (2019*c*). *Phys. Rev. Accel. Beams*, **22**, 122802.

[bb23] Stetsko, Yu. P. & Chang, S.-L. (1997). *Acta Cryst.* A**53**, 28–34.

[bb24] Tang, Z., Zheng, L., Chu, S., An, P., Huang, X., Hu, T. & Assoufid, L. (2021). *J. Appl. Cryst.* **54**, 976–981.

